# Erdheim-Chester disease with isolated neurological involvement

**DOI:** 10.1590/0100-3984.2016.0218

**Published:** 2018

**Authors:** Bruna Melo Coelho Loureiro, Albina Messias Altemani, Fabiano Reis

**Affiliations:** 1 Universidade Estadual de Campinas (Unicamp), Campinas, SP, Brazil.

*Dear Editor,*

**Figure 1:**
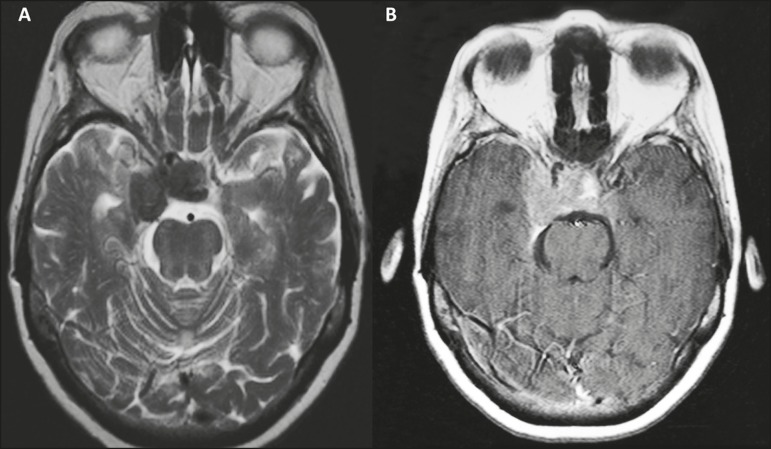
Magnetic resonance imaging scan showing a well-defined, compact, lobulated expansile lesion, measuring 3.0 × 1.5 × 3.0 cm, located in the right sellar and juxtasellar region, invading and occupying the sella turcica and the right juxtasellar region, with a hypointense signal on a T2-weighted image (**A**) and intense enhancement on a T1-weighted image acquired after gadolinium contrast administration (**B**). Note the reduction in the caliber of the intracavernous carotid arteries. The lesion is compressing and dislocating the optic chiasm anteriorly, cavitating the right medial temporal region, surrounding the pituitary gland, and extending to the suprasellar cistern. Anteriorly, it reaches the right optic canal.

A 25-year-old female patient presented with a seven-month history of progressive dysphagia, dysphonia, diplopia, ptosis of the right eyelid, weight loss, and sporadic pulsatile headache on the right side of the face. She had a history of hypertension, diabetes, unspecified thyroid disease, and smoking. The physical examination revealed satisfactory general health, although the patient was found to be malnourished, as well as to have deficits in the right third, fifth, and sixth cranial nerves. Magnetic resonance imaging (Figure 1) showed an expansile lesion located in the right sellar and juxtasellar region. A transsphenoidal biopsy was performed. The pathology and immunohistochemical study showed xanthomatous macrophages, together with CD 68 positive and CD1A negative histiocytes, consistent with a diagnosis of Erdheim-Chester disease. Computed tomography of the chest and abdomen showed no abnormalities.

Erdheim Chester disease is currently considered a clonal disorder, the pathogenesis of which is mediated primarily by a chronic, uncontrolled inflammatory process^(^^1^^)^. The Th1-type immune response involves activation of the following cytokines: IFN-α, IL-1/IL-1Ra, IL-6, IL-12, and MCP-1/CCL2. In studies of Erdheim-Chester disease, the most commonly reported gene mutation is that occurring in the BRAF V600E gene, which is seen in 57-75% of patients diagnosed with the disease. Mutations have also been reported in the MAPK (NRAS and MAP2K1) and PIK3 (PIK3CA) pathways^(^^2^^)^.

Histopathologically, Erdheim-Chester disease is a non-Langerhans cell histiocytosis, characterized by numerous macrophages with xanthomatous cytoplasm and small nuclei, together with giant cells, as well as few lymphocytes and eosinophils. The histiocytes are positive for CD-68, negative for S-100 protein, and negative for CD1A. It is noteworthy that Langerhans cells are positive for CD1A, negativity for CD1A therefore ruling out a diagnosis of Langerhans cell histiocytosis^(^^3^^)^.

**Figure 2:**
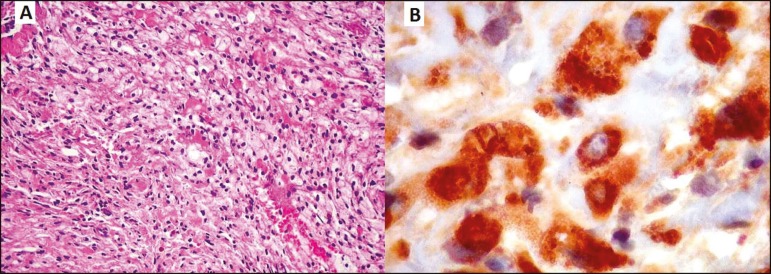
Non-Langerhans cell histiocytosis of the skull base. **A:** Photomicrograph showing abundant xanthomatous macrophages, in a solid arrangement, with small, dense nuclei and clear cytoplasm with lipid droplets. The cytoplasmic boundaries were more or less defined, depending on the area. **B:** Photomicrograph showing positivity for the macrophage marker CD68, which was the main antigen demonstrated in the lesion.

Clinically, Erdheim-Chester disease manifests as a systemic disease, involving bone, as well as the central nervous system (CNS), eyes, lungs, mediastinum, kidneys, and retroperitoneum^(^^4^^)^. The most common symptoms are bone pain accompanied by progressive weakness, especially in the lower limbs, together with fever, weight loss, exophthalmos, dyspnea, and signs of neurological impairment such as diabetes insipidus.

A recent extensive systematic review of 331 articles, including a collective total of 448 patients diagnosed with Erdheim-Chester disease, showed that neurological involvement was present as an initial manifestation in 25% of the patients and over the course of the disease in 50%^(^^5^^)^. Exophthalmos, other eye disorders, diabetes insipidus, cerebellar syndromes, seizure, and radiculopathy were the most commonly observed CNS manifestations. The most common features seen on imaging examinations were retro-orbital masses, involvement of the cerebellar dentate nucleus and meningeal lesions of the dura mater, as well as areas of cerebellar and brain stem demyelination. Suprasellar and infundibular lesions were more often accompanied by diabetes insipidus, hypopituitarism, and hyperprolactinemia. Involvement of the spinal cord was less common than was involvement of the brain and brain stem^(^^5^^)^.

In the present case, the neurological impairment was isolated. In the literature, we found no other reports of exclusive involvement of the CNS. The gender and age of our patient were also uncommon, given that the prevalence of Erdheim-Chester disease is highest among male patients between the 5th and the 7th decades of life^(^^5^^)^.
